# Walking Cadence during Moderate-Intensity Physical Activity in Pregnant Women

**DOI:** 10.3390/ijerph18126593

**Published:** 2021-06-19

**Authors:** Mallory Marshall, Beth Birchfield, Rebecca Rogers, Joyeuse Senga, McKenna Persch, Madison Currie, Daphne Schmid, Christopher Ballmann

**Affiliations:** Department of Kinesiology, Samford University, Lakeshore, AL 35229, USA; mmarshal@samford.edu (M.M.); cbirchfi@samford.edu (B.B.); rrogers1@samford.edu (R.R.); jsenga@samford.edu (J.S.); mpersch@samford.edu (M.P.); madi.rae.currie@gmail.com (M.C.); dschmid@samford.edu (D.S.)

**Keywords:** steps, metabolic equivalents, trimester, oxygen consumption, walking speed

## Abstract

Evidence has established that a cadence of 100 steps/min is indicative of the moderate intensity threshold of 3 metabolic equivalents (METs), but this has only been described in non-pregnant individuals. As metabolic alterations are well established during pregnancy, the purpose of this study was to determine if the walking cadence equivalent to 3 METs in pregnant women is similar to non-pregnant populations. Pregnant females (*n* = 29; age = 30.3 ± 3.2 years, gestational age = 23.9 ± 6.6 weeks) in their second or third trimester (>12 weeks gestation) completed three stages of treadmill walking for 5 min at different standardized walking speeds: 2.5, 3.0, and 3.5 miles per hour (mph). Oxygen consumption (VO2) and heart rate (HR) were measured each minute and METs were calculated for each stage. Real-time continuous monitoring of walking cadence was evaluated by an OptoGait gait analysis system. Following the three standardized speed stages, participants completed an additional stage walking at a speed that elicited 100 steps/min; VO2 and HR were also collected. A one-sample t-test was used to compare MET values at each stage to the heuristic 3 MET cutoff, and Pearson correlation coefficient was calculated to evaluate the relationship between cadence and METs. Mean cadence increased linearly across the three stages (2.5 mph = 103.7 ± 4.5, 3.0 mph = 112.5 ± 5.3, and 3.5 mph = 120.4 ± 6.2 steps/min), as did METs (2.5 mph = 2.7 ± 1.7, 3.0 mph = 3.2 ± 0.8, and 3.5 mph = 4.3 ± 1.8 METs) regardless of trimester. The average treadmill speed at which women walked at 100 steps/min was 2.4 ± 0.4 mph which elicited an oxygen consumption of 9.5 mL•kg^−1^•min^−1^, or 2.7 ± 0.7 METs. There was no significant difference between METs at 3.0 mph and the conventional 3 MET cut point for moderate-intensity PA (*p* < 0.05). There was a moderate and significant relationship between METs and cadence (2nd trimester: r = 0.51; 3rd trimester: r = 0.42). Current data indicate for the first time that the traditionally used 3 MET cutoff for moderate-intensity activity is appropriate for pregnant women despite metabolic alterations associated with pregnancy. This may have important implications for exercise prescription in pregnant populations.

## 1. Introduction

Walking cadence (steps/min) has been shown to effectively predict moderate-intensity (i.e., 3 MET) physical activity (PA) in the general population as well as in older adults. Specifically, the heuristic (i.e., practical, rounded) value of 100 steps/min corresponds well to moderate-intensity PA [1,2,3,4,5,6]. Measurement of PA intensity is important during pregnancy due to the many known health benefits of regular PA during pregnancy for both the mother and the fetus [7]. Walking is the most commonly utilized form of PA during pregnancy [8,9], and has been shown to reduce possible risk factors of gestational diabetes [10,11], preeclampsia [12], and excessive gestational weight gain [13], and increase the likelihood of healthy birthweight for the offspring [14,15]. However, perceptions of walking intensity may be altered as pregnancy progresses [16,17] due to many factors, including weight gain and altered center of gravity. Currently, the cut points for moderate-intensity PA for pregnant individuals are extrapolated from those used for general healthy non-pregnant populations. Further research is needed to determine if PA intensity thresholds are similar between these populations and could hold important implications for optimizing PA recommendations during pregnancy. 

During pregnancy, myriad metabolic and cardiovascular alterations occur, including elevated resting and submaximal heart rates [18], absolute rates of oxygen consumption, and increased cardiac parameters such as cardiac output, stroke volume, and blood volume [18,19,20,21]. Though there is conflicting evidence regarding gait changes across pregnancy, increases in weight gain and abdominal girth may result in functional consequences of movement such as shifts to center of gravity as well as altered gait patterns [22,23]. Whether these elevations in metabolism that are expected with pregnancy affect how walking cadence corresponds to oxygen consumption (measured in METs), however, is unknown. Use of cut offs that do not correspond well to VO2 during pregnancy may result in inappropriate PA recommendations for pregnant women. Thus, the purpose of this study was to determine if the walking cadence equivalent to three METs in pregnant women is similar to non-pregnant populations. 

## 2. Materials and Methods

### 2.1. Participants

A convenience sample of *n* = 19 2nd-trimester (14–26 weeks gestation; mean of 19.9 ± 4.1 weeks) and *n* = 10 3rd-trimester (27–40 weeks gestation; mean 31.5 ± 2.1 weeks) pregnant women were recruited via word of mouth, social media posts to the university neighborhood social network groups, and emails to the university community to participate in this study. Mean age was 30.3 ± 3.2 years. Eligible participants were at least 21 years of age and self-reported low-risk singleton pregnancies, no smoking, and no problems related to respiratory, cardiac, or musculoskeletal health. This study was approved by the university Institutional Review board prior to commencement of data collection, and written informed consent was provided by all participants.

### 2.2. Procedures

Participants reported to the laboratory and were fitted with a Polar heart rate monitor (Polar H7, Polar Electro, Bethpage, NY, USA) to continuously measure heart rate throughout the exercise session. In addition, expired gases were collected via Parvo Medics TrueOne 2400 (Parvo Medics, Sandy, UT, USA) metabolic measuring system over the course of the visit. After a 5-min rest period, participants walked on a motorized treadmill (Woodway 4Front, Woodway, INc., Waukesha, WI, USA) while gait parameters, including cadence, were assessed via the OptoGait analysis system (Microgate, Mahopac, NY, USA) for three different standardized speed stages: 2.5, 3.0, and 3.5 mph. Each stage lasted for a total of 5 min. Participants warmed up on the treadmill at a self-selected pace, while wearing the Parvo mask, to familiarize to the equipment prior to data collection. Oxygen consumption (VO_2_), heart rate (HR), and cadence (measured by OptoGait) were recorded each minute. Following another seated 5-min rest period, participants walked at a speed that elicited a cadence of 100 steps/min. The speed of the treadmill was adjusted until cadence reached +/−1 step/min of the 100 steps/min threshold and then participant maintained that speed for 2 min while VO2 and HR were recorded. Because reaching the 100 steps/min cadence took a minimum of three minutes for each participant, only two minutes of walking at this stage was required to measure a steady state HR and VO2.

### 2.3. Data Analysis

All data were analyzed using Excel (Microsoft, Redmond, WA, USA) and SPSS (Version 26, IBM Corp., Armonk, NY, USA) software. An analysis of variance (ANOVA) test was used to compare METs and HR recorded at the three walking speeds, and independent t-tests were used to compare 2nd- to 3rd-trimester women. For all analyses, was alpha set to 0.05. A one-sample t-test was used to compare measured MET values during walking to 3 METs, the heuristic value widely accepted as indicative of moderate intensity in the general population [24]. Finally, Pearson correlations were used to calculate the degree of association between METs and cadence. Correlations ranging between 0.1 to 0.39 were considered weak, 0.4–0.69 moderate, 0.7 to 0.89 strong, and 0.9–1.0 very strong [25].

## 3. Results

**Figure 1:**
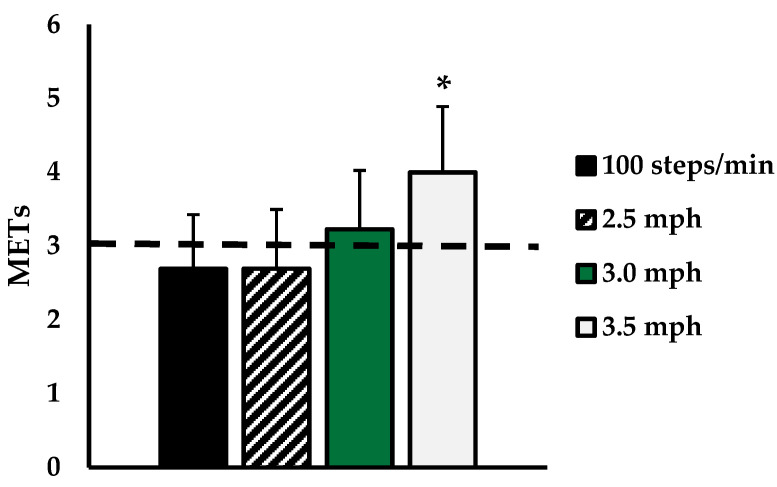
METs corresponding to 100 steps/min (Black), 2.5 mph (striped), 3.0 mph (green), and 3.5 mph (grey). Dashed line indicates 3 METs threshold for moderate-intensity exercise. Data are presented as mean ± SD. * indicates significantly different from 3 METs (*p* < 0.05).

As expected, HR and VO2 increased linearly with increased walking speed. In addition, HR was higher in 3rd-trimester compared to 2nd-trimester women during each walking stage, though the only statistically significant difference was at 2.5 mph. VO2 was not different between trimesters at any walking speed, as seen in Table 1. Because there were no differences in VO2 by trimester, all MET data are presented overall, with *n* = 29 2nd- and 3rd-trimester women represented. METs increased with intensity, but METs at 3.0 mph was not significantly different from 3 METs when compared using a one-sample t-test (*p* = 0.144). METs. As shown in Figure 1, METs at 3.5 mph were significantly greater than the 3 MET heuristic (*p* < 0.05). Figure 1 also shows the average METs at a stepping cadence of 100 steps/min, which was below 3 METS (2.7 ± 0.7 METs) but was not significantly different from 3 METs.

**Figure 2:**
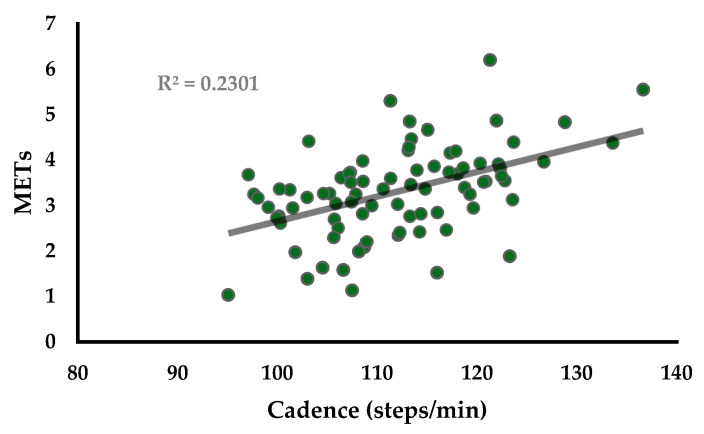
Scatterplot showing relationship between METs and Cadence (steps/min) for *n* = 29 pregnant women at three walking intensities (r = 0.48).

A moderate and statistically significant (r = 0.48; *p* < 0.01) correlation between cadence and METs was found in the sample, as seen in Figure 2. The correlation was slightly stronger in the 2nd (r = 0.51) than in the 3rd trimester (r = 0.42), though both were statistically significant (*p* < 0.05).

## 4. Discussion

Overall, we found that 3 METs is an appropriate heuristic measure of 100 steps/min, moderate-intensity PA in pregnant women, regardless of whether they were in the 2nd or 3rd trimester of pregnancy. Tudor-Locke et al. evaluated walking cadence at various treadmill speeds in 21–40-year-old adults (which is similar to the age group we analyzed, though we studied females only) and found similar VO_2_ and cadence values [1]. For example, at 3.0 mph, they reported an average relative VO_2_ of 14.2 mL/kg*min and 113.6 steps/min, while at the same treadmill speed, pregnant women averaged a VO_2_ of 11.3 mL/kg*min and cadence of 112.5 steps/min. Whether these slight differences can be attributed to the inclusion of males in one study or pregnancy status of the women in our analysis is not known, but regardless, 3 METs is still not significantly different, per results of a one-sample t-test, from METs measured at 3.0 mph or from METs measured at exactly 100 steps/min in our study.

We also found a moderate and significant correlation between stepping cadence and METs in pregnant women; this was expected and has been shown in non-pregnant populations of various ages [3,4,26], though correlations in our study were somewhat weaker than those shown by others in non-pregnant adults. We were surprised, however, by the finding that pregnant women did not have higher relative VO_2_ than 3 METs at a walking cadence of 100 steps/min; we expected that 3 METs was too low of a cut point for this population due to their gestational weight gain and presumed increase in metabolic rate due to pregnancy. During pregnancy, resting VO_2_ is elevated by around 6.5%, and HR/VO_2_ regression curves are flattened, suggesting a blunted HR response to exercise [27]. This may result in underestimation of energy expenditure during exercise. However, Dobson et al. found increased absolute, but not relative VO_2_ in late pregnancy compared to early [17], which supports the notion that walking does not elicit a significantly elevated metabolic rate in pregnant women compared to the non-pregnant state. While metabolism is indeed altered by pregnancy, the degree of change is insufficient to alter the 3 MET = moderate intensity heuristic, and thus this cut point can be used to prescribe exercise in this population as it can in typical healthy adults.

There are several strengths and limitations of the present study worth noting. Strengths include inclusion of both 2nd- and 3rd-trimester pregnant women in the analysis, as well as use of the OptoGait system, a validated measure of walking gait [28,29]. However, it should be noted that the sample chosen was a convenience sample and not designed to be representative of a generalized population of pregnant women. However, we have no evidence to suggest that the relationship between pregnancy metabolism and walking intensity varies by race, ethnicity, socioeconomic status, or other demographic factors not controlled for in the present study. Future research should, however, focus on recruitment of a larger sample representative of the general population of pregnant women, and also investigate the influence of factors that may influence metabolism (i.e., gestational weight gain, gestational age, maternal age) on the relationship between various cadences and METs. Finally, we did not collect data on physical activity or fitness levels of participants, which could potentially have affected the results if the sample were either highly active or inactive. However, other studies of cadence and METs in non-pregnant populations also did not control for fitness levels of participants, and no difference in HR/VO_2_ regression curves in exercising versus sedentary women was found in a study of metabolic changes across pregnancy [27].

## 5. Conclusions

A stepping cadence of 100 steps/minute can be used to elicit moderate-intensity PA and the associated benefits of that PA in pregnant women. There is no need to adjust the step cadence at which health benefits associated with moderate-intensity PA are achieved for this population.

## Figures and Tables

**Table 1:** Heart rate (HR), oxygen consumption (VO2), and cadence values in beats per minute, mL/kg*min, and steps/min, respectively, for 2nd- and 3rd-trimester pregnant women at three walking speeds.

		2.5 mph	3.0 mph	3.5 mph
2nd Trimester	HR	100.2 ± 13.7 *	113.2 ± 2	123.8 ± 21.1
	VO_2_	9.1 ± 3.4	11.5 ± 3.2	13.8 ± 3.5
	Steps/min	103.5 ± 4.4	112.0 ± 5.3	121.0 ± 6.5
3rd Trimester	HR	116.1 ± 17.5	125.7 ± 22.0	137.9 ± 23.8
	VO_2_	9.0 ± 2.3	10.9 ± 2.1	14.1 ± 2.1
	Steps/min	104.1 ± 4.8	112.9 ± 5.7	120.1 ± 6.0

* indicates significantly different from 3rd trimester (*p* < 0.05).

## Data Availability

All data are contained within this manuscript.
